# Hydrogel Microparticles for Bone Regeneration

**DOI:** 10.3390/gels10010028

**Published:** 2023-12-28

**Authors:** Cemile Bektas, Yong Mao

**Affiliations:** Laboratory for Biomaterials Research, Department of Chemistry and Chemical Biology, Rutgers University, 145 Bevier Rd., Piscataway, NJ 08854, USA; cemile.bektas@rutgers.edu

**Keywords:** hydrogels, hydrogel microparticles, microgels, tissue scaffolds, tissue engineering, bone regeneration, osteogenesis, cell delivery, bioactive-factor delivery, HMP-based scaffolds, HMP-incorporated scaffolds

## Abstract

Hydrogel microparticles (HMPs) stand out as promising entities in the realm of bone tissue regeneration, primarily due to their versatile capabilities in delivering cells and bioactive molecules/drugs. Their significance is underscored by distinct attributes such as injectability, biodegradability, high porosity, and mechanical tunability. These characteristics play a pivotal role in fostering vasculature formation, facilitating mineral deposition, and contributing to the overall regeneration of bone tissue. Fabricated through diverse techniques (batch emulsion, microfluidics, lithography, and electrohydrodynamic spraying), HMPs exhibit multifunctionality, serving as vehicles for drug and cell delivery, providing structural scaffolding, and functioning as bioinks for advanced 3D-printing applications. Distinguishing themselves from other scaffolds like bulk hydrogels, cryogels, foams, meshes, and fibers, HMPs provide a higher surface-area-to-volume ratio, promoting improved interactions with the surrounding tissues and facilitating the efficient delivery of cells and bioactive molecules. Notably, their minimally invasive injectability and modular properties, offering various designs and configurations, contribute to their attractiveness for biomedical applications. This comprehensive review aims to delve into the progressive advancements in HMPs, specifically for bone regeneration. The exploration encompasses synthesis and functionalization techniques, providing an understanding of their diverse applications, as documented in the existing literature. The overarching goal is to shed light on the advantages and potential of HMPs within the field of engineering bone tissue.

## 1. Introduction

The loss or dysfunction of skeletal tissue due to trauma, tumors, or abnormal development frequently requires surgical intervention to restore normal tissue function. Depending on the depth, type, and extent of the injury, the innate capacity of bones can be compromised or even inhibited, especially with large critical-sized defects [1]. The present clinical approaches to treating bone defects encompass both non-invasive and invasive therapies, with surgical intervention involving widely utilized bone grafts emerging as the predominant method [2]. However, numerous studies have highlighted the significant deficiencies, complications, and limitations of current clinical practices in bone repair and regeneration, including autografts and allografts [3,4,5,6]. Tissue-engineered bone grafts are a viable alternative treatment option that offers a uniform architecture and composition without potential donor-site morbidity or infection transmission. The traditional bone-tissue-engineering approach is composed of 3D scaffolds containing cells and growth factors. The success of tissue engineering hinges on the harmonious interaction and integration of cells and tissues using suitable cellular and physical cues [7]. Injectable materials, such as hydrogels, have been explored extensively in recent years as an alternative to invasive surgical procedures [8,9,10,11].

Hydrogels, which consist of crosslinked hydrophilic polymers forming intricate networks, represent a crucial class of materials in tissue-engineering applications. These remarkable materials have the capacity to absorb significant quantities of water, ranging from 10% to several times their own weight, resulting in pronounced swelling [12,13]. This swelling ability enables hydrogels to facilitate the transport of molecular and nano-scale substances within the material while maintaining solid-like mechanical properties. This exceptional characteristic, along with their versatility and similarity to the native extracellular matrix (ECM), has propelled the widespread adoption of hydrogels as scaffolding materials across various biomedical applications, including drug delivery [14,15], tissue engineering [16,17], and wound healing [18,19].

Typically, hydrogels are crosslinked to form bulk materials with dimensions at the millimeter scale. However, there are instances where micron-scale pore sizes are necessary, which can be achieved through various techniques, like electrospinning [20], cryogel formation [21], and porogen leaching [22]. Nevertheless, hydrogels may not always be the optimal choice, particularly when injection or smaller sizes are required, for example, in the case of irregularly shaped bone defects [23]. At the micron scale, hydrogels are referred to as microgels or hydrogel microparticles (HMPs), typically ranging from 1 to 1000 µm in size [24]. When the size of hydrogel particles is reduced to submicrometer ranges, they are referred to as nanogels [25]. Among other materials, HMPs emerge as promising scaffolds for bone regeneration, distinguished by their small size, injectability, and efficient encapsulation of live cells. Moreover, they offer modularity through mixing multiple HMP populations, all while providing significant porosity for cell support and proliferation [26,27,28].

This review will concentrate exclusively on the advancement of hydrogel microparticles for bone regeneration purposes, exploring synthesis and functionalization techniques, as well as highlighting the applications of HMPs for bone regeneration documented in the literature.

## 2. Fabrication of HMPs

### 2.1. Batch Emulsions

**Figure 1:**
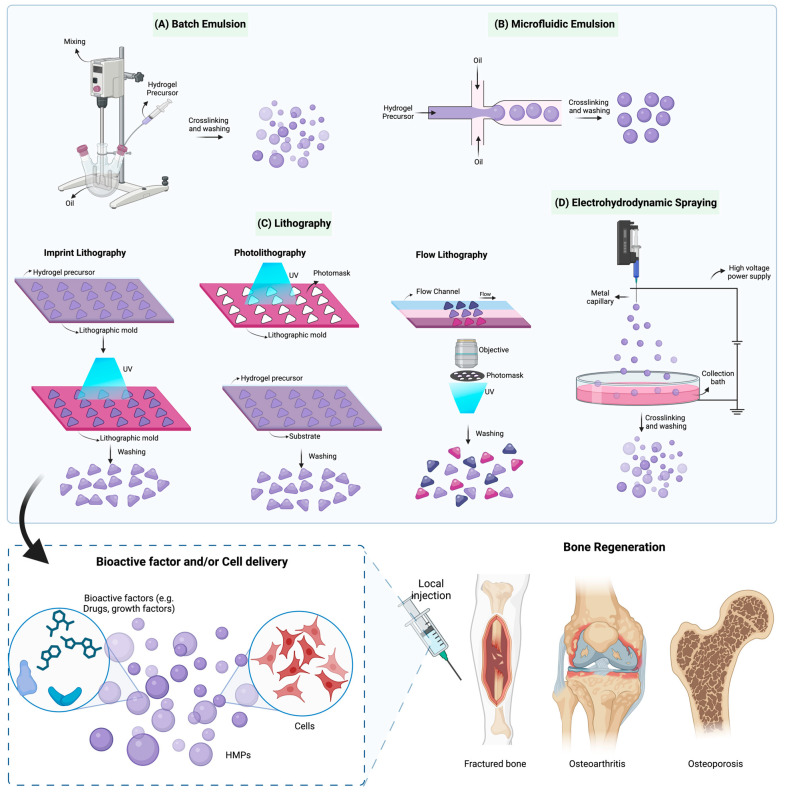
Schematic representation of HMP fabrication methods and their use in bone regeneration. Created with BioRender.com (accessed on 27 September 2023).

Batch emulsion, also known as inverse emulsion, employs the water-in-oil polymerization method, where simultaneously formed water-soluble droplets are evenly distributed within a continuous organic phase, facilitated by oil-soluble surfactants [29] (Figure 1A). The surfactant’s role is to hinder the re-aggregation of the droplets. Consistent formulations are obtained through mechanical agitation, where the extent and timing affect the dispersity and the size of the HMPs. After crosslinking, the oil phase is removed by subsequent washing steps, centrifugation, and filtration and optionally lyophilized for storage [30]. The crosslinking of the aqueous droplets can be achieved by adding a radical initiator to either phase and using various techniques, including photocrosslinking and thermal crosslinking [31]. Proteins/drug molecules or cells can be incorporated into the water phase during HMP formation if photocrosslinking [32] is used or can be loaded post-synthesis through the diffusion-based incubation of HMPs with a concentrated protein solution [33].

Initiators can be replaced by chemical crosslinkers, such as those involving gelatin microgels crosslinked with genipin or glutaraldehyde, also eliminating the need for a heating step and thermally initiated free-radical polymerization [33,34]. This method is compatible with biological molecules, as they can be loaded directly into the aqueous phase before the emulsification and polymerization steps with loading efficiencies of up to 98% [35,36,37] (Table 1).

The batch emulsion method offers several advantages, primarily its simplicity and potential for scalable synthesis, with limitations defined by the container size and the efficacy of emulsion mixing (Table 1). However, the inherent polydispersity in the HMPs produced through batch emulsion can lead to batch-to-batch variations unless production is precisely replicated in each reaction [38,39]. This polydispersity presents challenges when aiming for controlled drug release or cell encapsulation since it becomes challenging to regulate the quantity of a drug or the number of cells encapsulated in HMPs [38,40]. Furthermore, the release profile has been reported to differ between monodisperse and polydisperse HMPs, with monodisperse microparticles exhibiting a significantly reduced burst release compared to their polydisperse counterparts [41]. Collecting a specific size of HMPs can be achieved using sieves [42] or filters [43].

### 2.2. Microfluidic Emulsion

In contrast to the batch emulsion method, microfluidics offers several distinct advantages, as it allows for the precise control of the microparticle size and shape, facilitating the production of monodisperse populations with a wide range of shapes and compositions. HMPs are formed using droplet microfluidics in a junction geometry (such as T junction), usually fabricated using poly(dimethylsiloxane) (PDMS) via soft lithography [44,45] (Figure 1B). PDMS channels were produced from SU-8 molds, which were fabricated using photolithography techniques. Although alternative materials, such as glass, silicon, and thiolene, have been used in microfluidics, PDMS stands out due to its cost-effective manufacturing process and the ability to exert precise control over surface properties [46,47]. Methods to fabricate different types of microfluidic devices have been described in detail by Duffy et al. (1998) [46], Moreira et al. (2021) [47], Li et al. (2018) [48], and Lu et al. (2020) [49].

Microparticle formation using the microfluidic technique involves a continuous phase (usually oil), a surfactant to prevent the coalescence of the microparticles, and an aqueous hydrogel precursor phase. Droplets are generated due to the interaction of shear stress and interfacial tension forces between the different phases, and the HMPs are solidified either within or outside the microfluidic device using crosslinking methods such as photocrosslinking [50] or chemical or ionic crosslinking [51]. Following crosslinking, HMPs are collected and washed by centrifugation and stored in lyophilized form. The size and shape of the resulting particles can be adjusted by altering the flow rates, fluid composition, microfluidic device geometry, and channel size [47,48,52].

Microfluidic emulsions are versatile for encapsulating proteins, small molecules, and cells, achieving remarkable encapsulation efficiencies of up to 99% [53,54,55] (Table 1). The monodisperse characteristics of HMPs produced through this technique enable precise control over release profiles and the number of cells enclosed in each HMP. Microfluidics offers several significant advantages when it comes to controlling chemical reactions, including the precise management of factors such as heat and mass transport rates, a reduction in waste, and the ability to finely tune the characteristics of microparticles (Table 1). Moreover, it enables the production of composite microparticles by effectively combining various streams through the double emulsion technique [56,57]. Furthermore, microfluidics can seamlessly integrate with additional components, like heaters, coolers, sensors, and microscopy tools, significantly enhancing its utility in the realm of biological experiments [49].

The primary limitation of microfluidics lies in their comparatively low throughput when compared to batch emulsion methods [58]. This concern becomes particularly pronounced when generating small-diameter HMPs since the throughput tends to decrease with decreasing diameters [27]. To address this challenge, parallelized microfluidic designs incorporating multiple junctions within a single device have been employed [59,60]. However, it is crucial to design the channel geometry in these systems with meticulous intricacy, ensuring the effective management of pressure drops and flow fluctuations across the entire device.

### 2.3. Lithography

Lithographic processing methods, which enable the precise and reproducible templating of hydrogels, have emerged as appealing “top-down” alternatives to traditional approaches to synthesizing HMPs. Lithographic methods for producing HMPs can be categorized into three distinct groups: imprint lithography, photolithography, and flow lithography. While these techniques may vary in their format and specific processing steps, they typically adhere to a common workflow methodology. The initial step involves the preparation of a patterned template; then, the fluid reservoir is filled with the hydrogel precursor fluid(s), followed by the synthesis of HMPs through the concurrent processes of pattern transfer and crosslinking reactions, and the particles are recovered from the reservoir in the final step [61] (Figure 1C). Imprint lithography involves using a patterned mold, typically made of a polymer, to replicate the negative features of the particles to be generated. The master template, essential for this process, is created from a silicon wafer through soft lithography. This versatile technique offers the capability to produce microparticles in a wide range of shapes and sizes [62,63] (Table 1). Nonetheless, the complex nature of HMPs imposes practical limitations on the use of imprint lithography. Issues like the bending and buckling of the patterned template can hinder the successful replication of complex patterns, particularly those with intricate internal designs [61]. Photolithography and flow lithography both leverage a patterned photomask for the selective curing of hydrogel precursor regions, leading to the fabrication of HMPs [64]. In flow lithography, a flowing liquid is employed, with crosslinking taking place at regular intervals to form HMPs [61,65].

The major advantage of lithography techniques for forming HMPs is that the geometrical features can be well controlled and monodisperse particles can be obtained with up to 80% drug loading efficiency [62] (Table 1). These techniques also allow the formation of particles loaded with cells, and they do not involve any surfactant or oil to form the particles [63]. However, the main disadvantage of lithography methods is their low throughput compared to other techniques, where the size of the molds and the range of the light source are the main limiting factors [27,65].

### 2.4. Electrohydrodynamic Spraying

Electrohydrodynamic (EHD) spraying is a one-step HMP production system that does not require any additional solvents other than those used in the suspension itself [66]. In this system, a high electrical potential difference is applied to the flowing liquid by applying a voltage at the needle tip, breaking the hydrogel solution into small droplets, which are then collected in an earthed collection bath. Beyond a specific critical threshold, the electrical forces imposed become stronger than the surface tension that accumulates charges on the surfaces of the liquid droplets. Elevating the applied voltage further results in the creation of a conical-shaped liquid meniscus at the needle’s tip, referred to as a Taylor cone. Subsequently, this Taylor cone gives rise to a continuous jet that fragments into fine droplets upon reaching the collector’s surface, followed by crosslinking in the collecting solution [67,68] (Figure 1). The reaction is carried out at room temperature. Depending on factors such as the applied voltage, the inherent properties of the biomaterial, and the geometry of the needle, this technique enables the creation of intricate and complex structures with sizes as small as 1–2 μm [66,69] (Table 1). While achieving monodisperse particles can be challenging, it is possible to filter the particles to obtain a monodispersed particle distribution [27]. EHD spraying exhibits compatibility for delivering bioactive factors and cells, achieving encapsulation efficiencies of up to 99% [67,68,70,71] (Table 1).

Table 1 presents a comparison of various HMP fabrication techniques, highlighting their particle size, advantages, and disadvantages.

**Table 1:** Comparison of common HMP fabrication techniques.

Fabrication Method	Particle Size	Encapsulation Efficiency	Advantages	Disadvantages	References
Batch Emulsion	From a few micrometers to several millimeters	Up to 98%	Simple and easily scalable, compatible with wide range of materials	Batch-to-batch variations, limited control over the size of HMPs, uneven drug/cell encapsulation	[32,34,35,36,37,38,39]
Microfluidics	From a few micrometers to several millimeters	Up to 99%	Reproducible, well-controlled HMP size, even drug/cell loading, ease of production of composite HMPs, aseptic	Low throughput, time-consuming	[44,46,47,48,51,52,53,54,55,58]
Lithography	From a few micrometers to several hundred micrometers	Up to 80%	Control over size and shape, monodisperse particles, does not require surfactant or oil to form the particles	Low throughput, non-scalable, cost of photolithography masks	[61,62,63,64]
Electrohydrodynamic spraying	From a few micrometers to several hundred micrometers	Up to 99%	Simple, high encapsulation rate, no additional solvents	Difficult to control particle size and shape	[66,67,68,69,70,71]

## 3. Composition of HMPs

### 3.1. Natural Polymers

In the field of tissue engineering, a combination of stem cells, polymers, and various forms of physical, chemical, or biological stimuli is employed to establish a microenvironment that closely mimics in vivo conditions. To achieve this objective, both natural and synthetic polymers have been harnessed to create biocompatible and biodegradable polymeric structures. Natural polymers stand out among the options. They offer superior biocompatibility and biodegradability and pose no toxicity concerns [72]. These polymers feature numerous side groups along their molecular structure for further functionalization. Additionally, they often contain specific sequences, such as RGD (arginine–glycine–aspartic acid), as seen in gelatin, that enhance cellular behavior and improve cell adhesion properties [73]. While natural polymers frequently carry intrinsic cues for guiding stem cell behavior, a notable concern is the potential loss of their biological activity during processing or fabrication. This loss can not only affect their ability to direct stem cell fate but also lead to an immune response [74,75].

### 3.2. Collagen HMPs

Collagen, as the most abundant protein in the human body, plays a pivotal role in various biomedical applications, with type I collagen being the most prevalent and widely studied. Bone primarily comprises a mineral component, hydroxyapatite (60% of its composition), along with water (10%) and organic elements (30%), predominantly collagen (17–20%) [76,77]. Collagen’s remarkable properties, including its abundance, biocompatibility, low antigenicity, hydrophilicity, high porosity, and capacity for crosslinking, make it an ideal candidate for bone tissue engineering [74,78] (Table 2). The preparation of collagen HMPs often involves the widely used water-in-oil emulsification method. In this process, the hydrogel precursor is homogenized, leading to the formation of collagen droplets that undergo subsequent gelation, resulting in the creation of spherical collagen particles that can carry bioactive small molecules intended for bone regeneration [79]. By adjusting the crosslinking density or introducing electrostatic interactions between biomolecules and the collagen network, it becomes possible to fine-tune and control the kinetics of drug release from these particles [80,81]. In the literature, collagen HMPs have been used as vehicles for bioactive agents such as BMP-2 (bone morphogenic protein) and/or cells such as MSCs (mesenchymal stem cells) for bone tissue engineering. In their study, Khatami et al. employed the electrohydrodynamic spraying method to create collagen–alginate–nano-silica HMPs encapsulated with human-osteoblast-like MG-63 cells [82]. Their findings revealed that the incorporation of collagen into the HMPs led to a fourfold increase in cell proliferation and enhanced osteogenesis, as demonstrated by elevated levels of alkaline phosphatase activity and matrix mineralization. Additionally, the expression of osteocalcin and BMP-2 was substantially higher when compared to the control group (alginate + nano-silica), showing the osteoconductivity of the collagen HMPs. In another study, Chan et al. developed MSC-encapsulated collagen HMPs by forming 2.5 mL droplets directly in culture dishes, with gelation occurring at 37 °C under culture conditions (5% CO_2_) [83]. Their study revealed that collagen HMPs showed positive staining for alkaline phosphatase and calcium deposition, providing evidence for their osteogenicity; calcium phosphate deposition in the particles demonstrated their osteoconductivity; and the osteogenic differentiation of the MSCs showed the osteoinductive potential of the collagen particles.

Nonetheless, the utilization of collagen in HMP formation has been limited due to the challenges associated with collagen, such as poor mechanical properties, suboptimal processing conditions, and a propensity for denaturation during the particle formation process [73]. To address the mechanical limitations, collagen particles were prepared in blends with other materials, such as calcium phosphate [84] and chitosan [85]. Another issue related to collagen is its zoogenic sources, which may trigger immune reactions. The utilization of recombinant human collagen offers a robust alternative for fabricating HMPs that is safe, predictable, reproducible, and scalable [86,87].

#### 3.2.1. Gelatin HMPs

Gelatin, a denatured form of collagen, is a prominent material in biomedical applications due to its attractive biological properties and cost-effectiveness. It shares beneficial biological characteristics with collagen and is often used for medical or cosmetic purposes owing to its water solubility, nontoxicity, biocompatibility, and ability to promote strong cell adhesion [88]. Gelatin is particularly favored for its controllable degradation rates, its gentle gelling behavior, the ease of tailoring the crosslinking conditions, and the ease of functionalization and modification due to its diverse functional groups [89,90] (Table 2). Moreover, the production of gelatin HMPs is typically a straightforward process, often through batch emulsion [91] or microfluidic techniques [92]. The loading of bioactive molecules can be achieved by simple diffusion after the formation of gelatin particles, effectively separating the steps of crosslinking and drug loading [33]. This strategy offers the advantage of achieving controlled release kinetics by adjusting the crosslinking density and particle size before the introduction of active molecules. This approach protects the integrity of the loaded molecules, preventing any potential damage that might occur under harsh production conditions or during subsequent washing steps [91,93]. Moreover, gelatin offers significant advantages as a drug carrier, especially when loading charged biomolecules since the isoelectric point (IEP) of gelatin can be customized by selecting gelatin derived from either alkaline or acidic treatments [94].

Gelatin HMPs have found extensive applications in the field of bone tissue engineering. In their research, Fan et al. conducted a study in which they embedded mouse osteoblast MC3T3-E1 cells within gelatin HMPs [95]. The results indicated significantly higher cell viability and enhanced proliferation and differentiation capabilities, both in vitro and in vivo, as compared to cells cultured in gelatin hydrogels. In another study, a composite HMP consisting of gelatin and hydroxyapatite (G-HA) was fabricated through a wet-chemical method [96]. The results showed that G-HA composite particles enhanced the proliferation and differentiation of osteoblast-like cells. Moreover, in a rat calvarial defect model, the composite particles displayed superior osteoconductive and bioactive properties compared to both fibrin glue and Osteoset^®^ Bone Graft Substitute.

While gelatin offers desirable characteristics, it presents significant challenges, including the inability to load cells during processing and the utilization of toxic chemicals for crosslinking [97]. The diverse functional groups present in gelatin allow for the easy modification of its backbone. Notably, the methacrylation of amine groups, referred to as methacrylated gelatin (GelMA), emerges as a widely adopted technique among these modifications and facilitates rapid hydrogel formation under UV or visible light in the presence of a photoinitiator [97,98,99]. The versatility of GelMA has found extensive applications in tissue engineering, particularly in bone tissue engineering, where it is employed in hydrogel scaffolds and HMP production for cell and bioactive-factor delivery using a variety of the fabrication methods discussed above [100,101,102]. Another concern with gelatin is the risk of triggering immunogenic reactions due to its animal-derived origin, as in collagen. To address these limitations, recent efforts have focused on the creation of microspheres composed of recombinant gelatin with well-defined and tunable amino acid sequences, molecular weights, and isoelectric points [103].

#### 3.2.2. Alginate HMPs

Alginate, a naturally occurring polysaccharide found in brown algae and brown seaweed cell walls, has been widely used in tissue engineering and regenerative medicine due to its appealing properties, including biocompatibility, hydrophilicity, lack of immunogenicity, ease of cell encapsulation, and cost-effectiveness [104,105] (Table 2). The crosslinking of alginate hydrogel precursors is achieved using a diverse range of crosslinking agents, mainly calcium-based materials. When sodium alginate is placed into a bath containing divalent cations, such as Ca^2+^, the divalent cations replace sodium ions in the polymer, a process called ionotropic gelation [106]. These mild crosslinking conditions allow the encapsulation of sensitive cells and/or biomolecules into the alginate particles. For example, a recent study conducted by Kong and colleagues demonstrated the superior ability of MSCs encapsulated alginate HMPs and BMP-2-loaded polylactic acid (PLLA) to repair rat calvarial defects [107]. Their study showed that MSCs in Ca^2+^-crosslinked alginate HMPs retained their viability and potential to differentiate toward an osteogenic lineage, while BMP-2-loaded PLLA particles supported the differentiation of the encapsulated MSCs in vitro and osteogenesis in vivo by displaying a sustained release profile.

Despite their numerous advantages, alginate HMPs do present limitations, notably in terms of cell adhesion due to the absence of cell adhesion sites and slow degradation rates. However, chemical and physical modifications of alginate enable the fine-tuning of its properties, such as biodegradability, gelation properties, mechanical strength, and cell-binding affinity [108]. For example, the modification of alginate with growth factors such as BMP-2 and/or peptides such as RGD have been reported for bone tissue regeneration [109,110]. For instance, Moshaverinia et al. harnessed RGD-modified alginate HMPs to encapsulate gingival mesenchymal stem cells (GMSCs) and assessed their bone regeneration ability in vitro and in vivo in mice [111]. Their findings demonstrated a significant enhancement in the viability and osteogenic differentiation of MSCs in vitro and in vivo when encapsulated in RGD-modified alginate HMPs. This enhancement was evidenced by the expression of osteogenic markers, including Runx2 (Runt-related transcription factor 2), ALP (alkaline phosphatase), and osteocalcin in vitro, coupled with the effective repair of critical-sized calvarial defects in mice in vivo. These findings highlight the potential of RGD-modified alginate HMPs in accelerated craniofacial bone tissue regeneration.

#### 3.2.3. Chitosan HMPs

Chitosan, a natural biopolymer derived from chitin, is widely studied for bone regeneration applications owing to its remarkable characteristics, including its biocompatibility, biodegradability, ease of processing, and antibacterial nature (Table 2). Most of these distinctive characteristics are attributed to the primary amines found along the chitosan structure. Through these amino groups, chitosan has the capacity to be functionalized or establish ionic complexes with a wide range of anionic species, such as synthetic polymers like poly(acrylic acid), proteins, lipids, and DNA [112]. The degradability of chitosan is modulated by its amino groups and its polysaccharide composition, which incorporates glycosidic bonds that can be broken down in vivo by various proteases, primarily lysozyme [113].

Chitosan-based HMPs are manufactured through a variety of techniques [114,115,116,117], with batch emulsion being the most commonly employed method [73]. For example, Cai et al. fabricated chitosan HMPs with either positive charges to carry osteoinductive BMP-2 or negative charges to load an antibacterial factor, berberine (Bbr), using the batch emulsion method [118]. Their studies demonstrated stimulated osteogenic differentiation, enhanced in vivo bone reconstruction, and high antibacterial activity in a sustainable manner, showing the efficacy of chitosan HMPs in promoting bone healing while preventing bacterial infections. Nevertheless, chitosan on its own exhibits suboptimal mechanical properties. The crosslinking of chitosan with diverse crosslinkers, such as glutaraldehyde [119], genipin [120], and glyoxal [121], enhances its mechanical strength and extends the delivery duration of biological molecules. The incorporation of bioceramics [117], proteins [122,123], or polymers [124] also augments chitosan’s effectiveness in bone regeneration. For instance, Wise and colleagues combined chitosan with collagen and successfully encapsulated MSCs within chitosan–collagen HMPs using the batch emulsion method [123]. Following the pre-differentiation of MSCs toward the osteogenic lineage, they introduced these particles into a mouse calvarial defect model. Combining them with collagen and employing a pre-differentiation strategy with the HMPs significantly enhanced the repair of bone defects compared to undifferentiated control cells.

### 3.3. Synthetic Materials

Synthetic materials are attractive alternatives to natural materials, offering numerous advantages. Many synthetic materials are non-immunogenic, biocompatible, and biodegradable; possess greater mechanical and chemical stability; are reproducible with minimal batch-to-batch variation; and can be modified easily. Their mechanical properties and degradation rates can be finely tuned through adjustments in production and processing conditions [12]. Furthermore, unlike their natural counterparts, synthetic materials are not constrained by limited sources. Despite these advantages, the exploration of synthetic hydrogels for microencapsulation remains relatively limited in the literature due to the harsh processing conditions involved, such as nonphysiological pH, high temperatures, or the use of organic solvents. These conditions are often not compatible with sensitive cells and proteins. Among the synthetic polymers, poly(ethylene glycol) (PEG) and poly(vinyl alcohol) (PVA) have been employed in the fabrication of HMPs for bone regeneration.

#### 3.3.1. Poly(ethylene glycol) (PEG) HMPs

PEG, an FDA (Food and Drug Administration)-approved polymer, is one of the extensively studied synthetic polymers for hydrogel formation in tissue engineering due to its hydrophilicity, biocompatibility, and nontoxicity [125,126] (Table 2). Various methods, including EHD spraying [127], lithography [128], batch emulsion [129], and microfluidic emulsion [130], are employed to produce PEG hydrogel microparticles (HMPs). In the case of acrylated or methacrylated PEG HMPs, crosslinking takes place in an aqueous environment through UV polymerization in the presence of a suitable photoinitiator [130]. In a notable study, Sonnet et al. synthesized poly(ethylene glycol) diacrylate and microencapsulated Wistar skin fibroblasts transduced by adenovirus-expressing BMP2 [131]. The fabrication of HMPs was facilitated by a hydrophobic photoinitiator (2,2-dimethoxy-2-phenyl acetophenone in 1-vinyl-2-pyrrolidinone), allowing for white-light crosslinking during batch emulsion. Their study showed the complete healing of a 5 mm long femur defect in a rat model in less than 3 weeks, achieved with 100-fold lower levels of BMP-2 protein compared to various doses of recombinant BMP-2 protein, which required 2–3 months for healing.

Despite its advantages, the slow degradation rates of PEG in body fluids and its resistance to protein and cell adhesion limit its applicability. To address the slow degradation issue, one approach is to modify the degradation rate of PEG by incorporating degradable monomers like poly(propylene fumarate) (PPF) or integrating degradable terminal cyclic acetals [132]. Additionally, the resistance of PEG to cell adhesion can be overcome by incorporating cell-adhesive peptide sequences such as RGD, which has been demonstrated to significantly enhance MSC viability [133,134].

#### 3.3.2. Poly(vinyl alcohol) (PVA) HMPs

PVA, obtained from the partial hydrolysis of poly(vinyl acetate), is another widely used synthetic polymer in HMP preparations because of its biodegradability, biocompatibility, high swelling ratio, and FDA approval for oral medicine [44,135] (Table 2). PVA-based HMPs/hydrogels have been reported for cell encapsulation and enzyme immobilization, often produced using microfluidic [136] or emulsion [137,138] methods. In their study, Hou et al. utilized high-throughput microfluidic technology to develop injectable, degradable HMPs based on poly(vinyl alcohol) (PVA) incorporating MSCs and BMP-2 [139]. The tunable mechanical and degradability properties of the polymer backbone were achieved through the functionalization of PVA with vinyl ether acrylate (VEA) and thiol (SH) groups. Their findings revealed that encapsulating BMP-2 into the particles along with MSCs enhanced osteogenic differentiation, which was evidenced by a significant increase in ALP activity, calcium content, and the expression levels of RunX2 and OPN (Osteopontin) genes. While PVA, like other neutral hydrogels, may not be inherently attractive for cells and proteins [13,135], the fabrication of composite HMPs consisting of PVA with hydroxyapatite [140,141], proteins [142], or other natural materials [143] has been studied to enhance bioactivity.

Table 2 provides an overview of the biomaterials employed in the fabrication of HMPs by comparing their respective advantages and disadvantages.

**Table 2:** Summarized comparison of biomaterials in HMP fabrication.

Biomaterial Classification	Biomaterial	Advantages	Disadvantages	HMP Fabrication Techniques	References
Natural	Collagen	Biocompatible, degradable, good bone conduction activity	Poor mechanical features, suboptimal processing conditions, risk of denaturation during processing	Batch emulsion, EHD spraying	[73,74,78,79,144]
	Gelatin	Biocompatible, nontoxic, tunable degradation, tailored crosslinking conditions, ease of functionalization and modification	Risk of triggering immunogenic reactions	Batch emulsion, microfluidics, EHD spraying, lithography	[88,89,90,91,92,93,145]
	Alginate	Biocompatible, lack of immunogenicity, cost-effective, gentle crosslinking, tunable mechanical properties	Lack of cell adhesion sites, slow degradation	Microfluidic emulsion, EHD spraying, batch emulsion	[100,104,106,107,108,109,146]
	Chitosan	Biocompatible, ease of processing, antibacterial nature, tunable degradation rates	Suboptimal mechanical properties, batch-to-batch variation	Batch emulsion, microfluidics, EHD spraying	[112,113,114,115,116,118]
Synthetic	Poly(ethylene glycol) (PEG)	Biocompatible, nontoxic, ease of functionalization and modification	Slow degradation rates, resist protein and cell adhesion	Batch emulsion, microfluidic emulsion, lithography, EHD spraying	[125,126,127,128,129,130,133,134]
	Poly(vinyl alcohol) (PVA)	Biodegradable, biocompatible, FDA approved, ease of functionalization	Lack of cell adhesion sites	Microfluidics, batch emulsion, lithography	[44,135,136,137,138,147]

## 4. Applications of HMPs for Bone Regeneration

The unique properties of HMPs mentioned above make them attractive for numerous biomedical applications, as well as bone tissue regeneration. This section explores several applications, such as the delivery of bioactive factors and cells, scaffold building with HMPs, and their incorporation as reinforcing materials into scaffolds.

### 4.1. Bioactive-Factor Delivery

**Figure 2:**
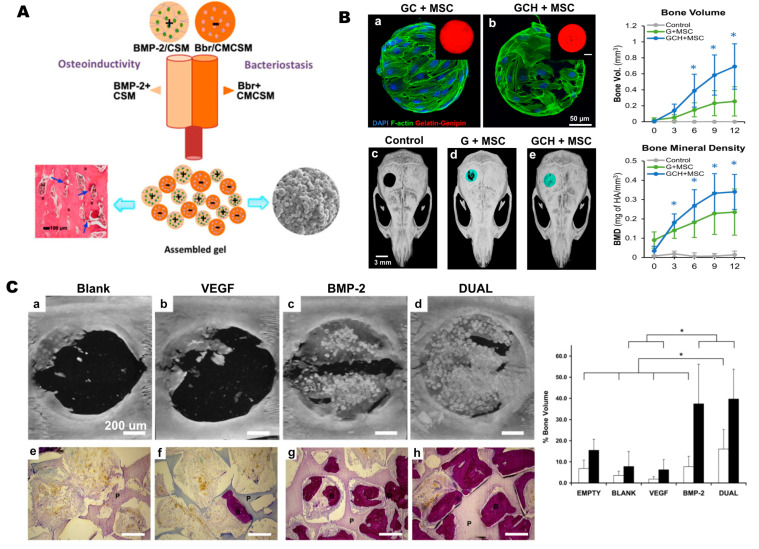
Applications of HMPs in bioactive-factor delivery. (**A**) Schematic representation of oppositely charged chitosan (+) and O-carboxymethyl chitosan (−) HMPs loaded with BMP-2 and berberine, promoting osteogenic and antimicrobial activities, respectively. Reprinted from [118] with permission. Copyright 2018, American Chemical Society. (**B**) MSCs seeded in osteogenic HMPs composed of (**a**) gelatin and chitosan (GC) and (**b**) gelatin, chitosan, and hydroxyapatite (GCH) stained with DAPI (nuclei, blue) and F-actin (cytoskeleton, green). The insets depict the red fluorescence emitted by the gelatin matrix crosslinked with genipin. Composite HMPs exhibited the highest defect closure (**e**), bone volume, and bone mineral density (upper-right graphs) compared to control (**c**) and MSCs seeded in gelatin (G+MSC) (**d**). * *p* <0.05. Reprinted from [158] with permission. Copyright 2022, Nature. (**C**) Dual delivery of VEGF and BMP-2 from gelatin HMPs embedded in porous poly(propylene fumarate) scaffolds, demonstrating effective defect closure (**d**) compared to blank (**a**), containing only VEGF (**b**), and BMP-2 (**c**), resulting in significant bone formation in both the pores and along the scaffold surfaces (**h**), and the highest bone volume (bottom right graph) compared to blank (**e**), containing only VEGF (**f**), and BMP-2 (**g**), * *p* < 0.05. Scale bar is 200 μm. Reprinted from [156] with permission. Copyright 2008, Elsevier.

HMPs show significant promise in the field of bone regeneration for the delivery of bioactive factors, such as growth factors or small molecules, by protecting, transporting, and releasing these factors in a controlled manner [148]. This functionality addresses numerous limitations associated with conventional drug administration routes, including intravenous and oral methods, which typically require high doses and repeated administration and may lead to off-target effects [149]. Conversely, the small size of HMPs facilitates the transport of pre-engineered microtissues through small needles and catheters [150]. Additionally, HMPs offer remarkable versatility, as multiple HMP populations can be seamlessly combined, integrating diverse release profiles and degradation behaviors into a single injection [118] (Figure 2A). This adaptability holds promising advantages for tissue-repair strategies, aligning effectively with biological signaling cascades.

The water retention capacity, ECM-mimicking environment, biocompatibility, and degradability of HMPs make them an excellent host for bioactive factors, enabling their encapsulation and controlled release [151]. Among these factors, BMP-2 has attracted significant attention as an FDA-approved growth factor [152] and has been extensively studied in vitro, in vivo, and clinically for bone regeneration [131,153,154]. As a carrier HMP, natural, synthetic, and composite materials have been reported in the literature to carry BMP-2. For instance, gelatin-based HMPs created through the batch emulsion method were employed to encapsulate BMP-2, resulting in enhanced bone repair in vivo in osteoporotic goats after a 45-day implantation period [155]. The findings revealed increased bone mineralization when BMP-2 was delivered in encapsulated form within gelatin HMPs, in conjunction with bone cement, compared to BMP-2 administered solely with bone cement and without microgels. In another example, BMP-2 was encapsulated within chitosan microspheres and incorporated into collagen sponges for implantation into 15 mm radius defects in rabbits [85]. Remarkably, within just 4 weeks, the HMP-loaded scaffolds successfully bridged the defects, and the complete healing of the defects, accompanied by the recanalization of the bone marrow cavity, was achieved within 12 weeks. Mixed populations of HMPs have also shown promise in bone repair. In a study conducted by Patel and colleagues, vascular endothelial growth factor (VEGF) was utilized as an angiogenic factor, and BMP-2 was used as an osteogenic factor, both carried by gelatin microgels [156]. The dual delivery of VEGF and BMP-2 in a rat calvarial critical-size defect, measuring 8 mm in diameter, demonstrated enhanced bone repair, with increased osteoid secretion and mineralization compared to groups where either of the factors was used alone (Figure 2C). In addition to growth factors, the incorporation of bioceramics, such as hydroxyapatite, into the composition of HMPs stands out as a common and widely discussed approach in the literature. This method aims to mimic the natural ECM, enhancing the mechanical strength of the particles and augmenting their bioactive properties more accurately [157,158,159,160]. As an example, in their investigation, Patrick et al. presented an osteogenic microgel comprising chitosan, gelatin, and hydroxyapatite, designed to mimic the bone matrix [158]. This formulation supported robust cell attachment, proliferation, and MSC differentiation and, notably, exhibited successful bone regeneration in a critical-sized defect, achieving over 95% closure within 12 weeks (Figure 2B). This underscores the efficacy of multiphasic scaffolds with tissue-specific biochemical and biophysical properties.

### 4.2. Cell Delivery

**Figure 3:**
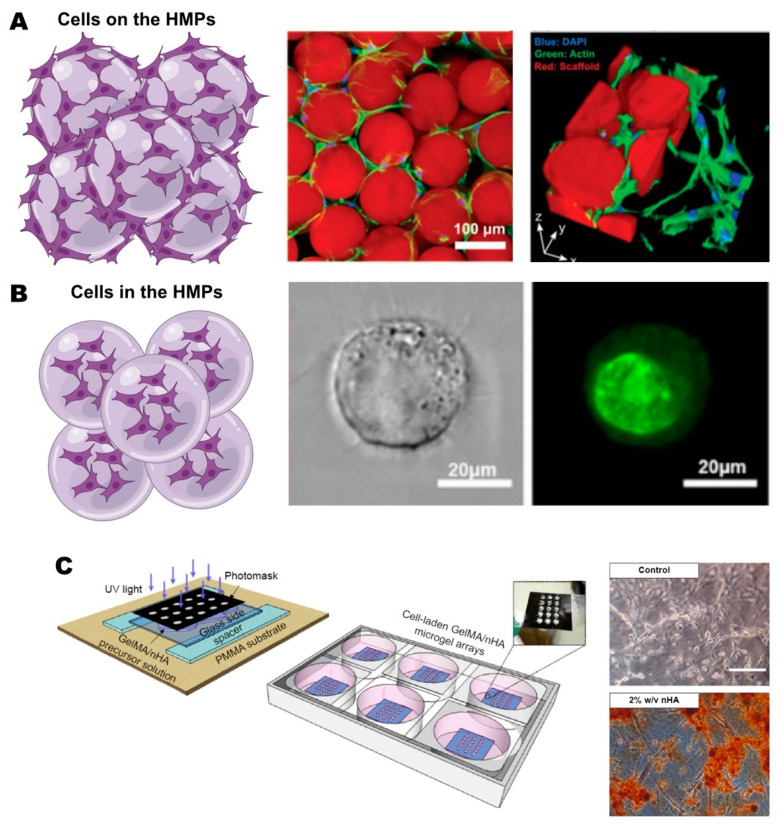
Applications of HMPs in cell delivery. (**A**) Delivery of the cells on the HMPs, stained with DAPI (nuclei, blue), actin (cytoskeleton, green). The red fluorescence is from scaffold. (**B**) Delivery of the cells in the HMPs, stained with Calcein-AM (Live cells, green). Reprinted from [164,167] with permission. Copyright 2019, John Wiley and Sons and 2020, Elsevier, respectively. (**C**) Schematic representation of GelMA/nHA HMP fabrication and representative images of cell-laden microgels with and without nHA after 10 days of osteoinduction, stained with Alizarin Red S staining for calcium deposition. Scale bar is 100 μm. Reprinted from [157] with permission. Copyright 2016, Dovepress. Schematics of (**A**,**B**) were created with BioRender.com (accessed on 27 November 2023).

Delivering cells to damaged and diseased tissues holds great potential; however, challenges such as limited cell survival and engraftment post-delivery have hindered progress in this field [161,162]. Consequently, there is growing interest in utilizing delivery vehicles to enhance cell integration, viability, and function. HMPs offer an efficient platform for encapsulating, growing, and delivering cells, with fabrication techniques like emulsion, electrohydrodynamic spraying, and microfluidics being compatible with cell encapsulation (Figure 3A). HMPs provide a substantial adhesion surface for cells, and those featuring surface pores enhance nutrient and oxygen diffusion [26,163].

The design of HMPs is crucial in optimizing their benefits, with the selection of material for cell embedding and the processing conditions for optimal cell encapsulation holding great importance. Both of these factors shape the properties of HMPs, encompassing degradation and mechanical strength, which are important for controlling the timing of cell delivery and achieving effective integration with the environment and the particles themselves [27]. In some cases, cell delivery occurs on the surface of HMPs instead of within them, enhancing nutrient and oxygen availability for the cells and maximizing their viability [24]. HMPs can undergo additional crosslinking to immobilize the particles and stabilize the packing configuration, referred to as microporous annealed particles (MAPs) [164] (Figure 3A).

HMPs have been extensively used to deliver MSCs for bone tissue repair. For example, Moshaverinia et al. co-encapsulated an anti-BMP2 monoclonal antibody and MSCs in RGD-coupled alginate HMPs by extruding droplets of an alginate mixture into a calcium chloride solution to achieve crosslinking [165]. The co-delivery system demonstrated enhanced osteogenic differentiation in vitro, evident through the elevated expression of key osteogenesis regulators, namely, Runx2 and Smad 1 (Suppressor of Mother against Decapentaplegic), and showed complete bone healing in critical-sized calvarial defects in mice in vivo. In a similar approach, MSCs and BMP-2 were encapsulated within GelMA-based HMPs employing microfluidic emulsion technology [166]. This approach showed the microgel system’s capability to sustain cell viability, promote cell migration, and induce cell differentiation in vitro, subsequently leading to robust bone regeneration in vivo.

By utilizing microfluidic technology, the continuous encapsulation of cells is also possible at the single-cell level. In a study by An et al., single-MSC encapsulation in alginate HMPs was achieved using microfluidic techniques [167] (Figure 3B). Their research demonstrated that these cell-laden microgels enhanced bone formation in a rat tibial model by creating a highly controlled osteogenic microenvironment compared to MSCs mixed with microgels.

A synergistic approach involving desired cell populations for promoting bone repair has also been documented in the literature. Wise et al. utilized pre-differentiated MSCs alongside freshly isolated Bone Marrow Mononuclear Cells (BMMCs) within collagen–chitosan HMPs [123]. A robust synergistic effect on volume and ectopic bone formation was observed after a 5-week implantation of microgels into a subcutaneous rat dorsum model, surpassing the outcomes achieved with either cell type alone. Similarly, Grellier et al. employed a comparable strategy by immobilizing human osteoprogenitors derived from MSCs alongside human umbilical vein endothelial cells (HUVECs) within RGD-modified alginate HMPs [110]. Their study revealed the upregulated gene expression of ALP and osteocalcin, along with increased mineralization deposits in the co-immobilized HMPs in vitro. Furthermore, in vivo studies conducted on bone defects in nude mice demonstrated significant bone mineralization in and around the sites when employing the co-immobilization approach.

To better mimic the native ECM of bone tissues and enhance the biological properties of materials, nanofeatures can be incorporated into HMPs. A popular approach involves the use of nanohydroxyapatite (nHAp), which has been shown to enhance osteoblast adhesion, proliferation, and differentiation by directly influencing biocompatibility, the specific surface area, and the surface roughness of the HMPs [168]. For example, Chen et al. fabricated periodontal ligament stem-cell-laden GelMA/nHAp microgel arrays (Figure 3C) and demonstrated promoted osteogenic differentiation by elevated levels of calcium deposition and osteogenic markers such as ALP, Runx2, OCN, and BSP [157]. Furthermore, in vivo experiments in nude mice revealed enhanced osteogenesis, characterized by the augmented formation of mineralized tissue and increased vascularization compared to the pure GelMA group.

### 4.3. Scaffold Design with HMPs

As mentioned earlier, HMPs find extensive applications in delivering bioactive factors or cells for diverse tissue-engineering strategies, including bone tissue regeneration. They can be delivered as standalone entities, utilized as fundamental building blocks to create comprehensive scaffolds, or employed as a dispersed phase enveloped by a continuous matrix. In this section, two primary scaffold designs for bone tissue engineering will be discussed: HMP-based scaffolds and HMP-reinforced scaffolds.

#### 4.3.1. HMP-Based Scaffolds

Scaffolds based on HMPs are crafted using a “bottom-up” strategy, which has gained significant popularity in recent years for its meticulous integration of biomaterials, cells, and biomolecules through the precise assembly of microgels as fundamental building blocks. The interstitial spaces between these particles, crucial for enabling cell ingrowth, facilitate continuous nutrient and oxygen transfer [169,170]. HMP-based scaffolds are constructed through random packing, directed assembly, and 3D bioprinting techniques.

**Figure 4:**
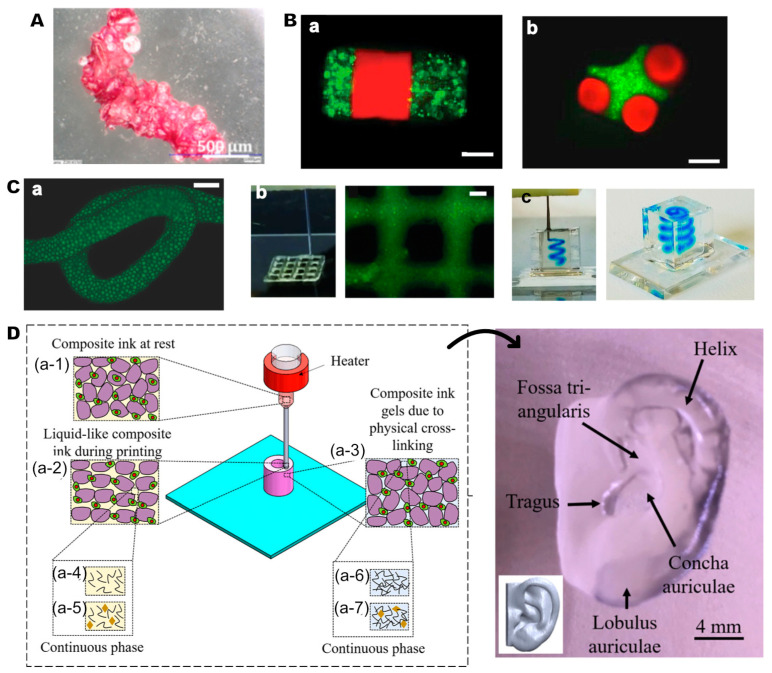
HMP-based scaffolds. (**A**) Auto-assembled MC3T3-E1-cell-laden collagen-coated PLGA microspheres in osteogenic media on Day 7, displaying positive staining for alkaline phosphatase. Reprinted from [171] with permission. Copyright 2021, MDPI. (**B**) Directed assembly of cell-laden PEGDA (polyethylene glycol diacrylate) microgels into (**a**) rod-shaped and (**b**) lock-and-key assemblies, stained with FITC-dextran (green) and Nile red (red). Scale bars 200 μm. Reprinted from [176] with permission. Copyright 2008, PNAS. (**C**) Jammed HMPs fabricated by 3D bioprinting, including (**a**) extruded filament of norbornene-modified hyaluronic acid HMPs, (**b**) 3D bioprinting on a platform, and (**c**) within a shear-thinning support transparent hydrogel reservoir. Reprinted from [182] with permission. Copyright 2018, John Wiley and Sons. (**D**) Schematic representation of direct printing of gelatin HMP-based composite bioink, demonstrating applicability for complex organ engineering, such as the human ear. Reprinted from [184] with permission. Copyright 2020, American Chemical Society.

In the random packing approach, HMPs are randomly assembled to create a 3D scaffold, optimizing the delivery of bioactive factors and cells to the target site. In a relevant study, Mielan et al. demonstrated the auto-assembly of collagen-coated poly(L-lactide-co-glycolide) (PLGA) microspheres seeded with preosteoblast MC3T3-E1 cells for bone regeneration [171]. Their findings revealed that collagen coating facilitated cell growth, proliferation, and osteogenic differentiation, with auto-assembly observed by day 7 (Figure 4A). However, due to the reliance on interparticle interactions, concerns arise regarding mechanical stability, potentially leading to the dissociation of individual particles from the defect site [73]. To address this issue, glues [172] and crosslinking agents [173] have been suggested in the literature to prevent microspheres from flowing out of the treatment site. For example, in a recent study, Nisal et al. introduced a unique bone regeneration approach utilizing silk fibroin microgels, randomly packed with the assistance of a dilute silk fibroin solution [174]. The resulting 3D HMP-based construct exhibited mechanical properties akin to native cancellous bone, demonstrating biocompatibility and supporting cell adhesion, proliferation, and differentiation in vitro. In vivo implantation in rabbit femurs revealed woven bone formation after just 4 weeks, highlighting the promising potential of this method. In random packing, the structure of the resulting 3D scaffold can be fine-tuned by incorporating various customized particles encapsulating biomolecules, bioceramics, or cells.

Creating HMP-based scaffolds through the direct assembly method involves utilizing cohesive forces such as hydrophobic interactions, magnetic forces, or electrostatic forces. In contrast to random packing, this approach enables the creation of microengineered tissues in a directed and scalable manner, providing precise control over the scaffold’s structure [175]. The directed assembly of cell-laden microgels for scaffold fabrication was initially documented in 2008 by Du et al. In their pioneering work, controlled assembly was achieved at the oil–water interface through mechanical agitation and manual manipulation using a pipette tip [176]. This innovative approach resulted in various structures, including linear (Figure 4B(b)), random, branched, offset, and lock-and-key-shaped (Figure 4B(c)) microgels. Subsequent to this groundbreaking study, numerous research groups further explored and applied this method, showcasing the controlled assembly of various HMP types into diverse shapes [177,178,179,180]. While many current studies are in the proof-of-concept stage, these advancements hold significant potential for various tissue-engineering applications, including bone tissue regeneration.

Three-dimensional bioprinting is a powerful technique for precisely designing 3D structures in personalized tissue engineering and regenerative medicine applications, utilizing computer-aided design (CAD) data for custom architectures. Among various 3D-printing technologies, like stereolithography, inkjet printing, or fused deposition modeling, extrusion bioprinting stands out due to its compatibility with hydrogels, offering cost-effectiveness and mild printing conditions [181]. Hydrogel precursor solutions, known as bioinks, require high viscosity to maintain structural integrity after extrusion, which can affect cell viability. Unlike the conventional hydrogel precursors utilized in bioprinting, microgels demonstrate shear-thinning behavior. They act as solids but exhibit fluidic collective movement under external forces, allowing for flow and rapid recovery upon deposition, thereby ensuring high cell viability [182,183]. As a proof of concept, Highly et al. demonstrated the utilization of cell-laden HMPs engineered from diverse materials, such as thiol-ene-crosslinked hyaluronic acid, photocrosslinked PEG, and thermosensitive agarose [182]. Following particle fabrication, they subjected them to “jamming” through vacuum-driven filtration, loaded them into a syringe using centrifugation, and proceeded with bioprinting. This innovative approach enabled them to bioprint customized structures without the need for additional materials or crosslinkers for stabilization (Figure 4C). However, given that this technique relies solely on physical interactions between particles, ensuring the long-term stability of the constructs poses a challenge. The inclusion of a carrier bioink material can enhance the stability of HMP-based bioprinted constructs [184] (Figure 4D). For example, in their research, Chai et al. designed core–shell HMPs with a collagen core layer and an alginate shell using a multichannel microfluidic device and blended them with GelMA and methacrylated silk fibroin (SilMA) hydrogel precursors for the fabrication of 3D-printed constructs [100]. The incorporation of cells into the microgels notably improved the survival rate of the stem cells. Upon subcutaneous implantation in Spraque-Dawley rats, the resulting constructs demonstrated enhanced bone formation compared to those without microgels, highlighting the efficacy of this technique.

#### 4.3.2. Reinforcing Scaffolds: HMP-Incorporated Scaffolds

**Figure 5:**
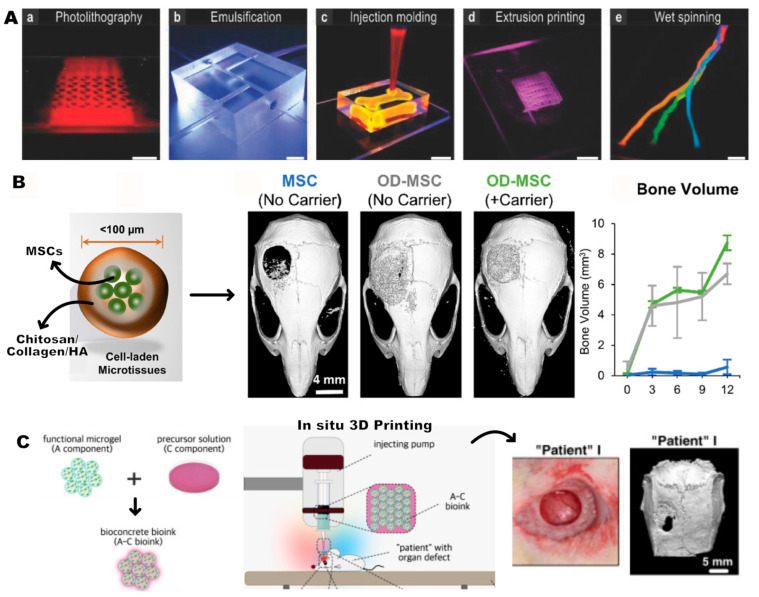
(**A**) Fabrication process of PEGDA HMPs using (**a**) photolithography, (**b**) emulsification, (**c**) injection molding collagen, (**d**) extrusion bioprinting alginate/GelMA, and (**e**) wet spinning with subsequent weaving of alginate. Scale bars 5 mm. Reprinted from [186] with permission. Copyright 2016, John Wiley and Sons. (**B**) MicroCT images depict the newly formed bone volume in the defect area treated with HMPs loaded with MSCs, pre-differentiated MSCs (Osteogenic Differentiated, OD), and pre-differentiated MSCs in a fibrin carrier, demonstrating complete healing with the carrier. Reprinted from [187] with permission. Copyright 2019, Elsevier. (**C**) In situ 3D printing of bioconcrete bioink (GelMA HMPs and GelMA cement) on the patient, illustrating effective bone formation within 6 weeks. Reprinted from [188] with permission. Copyright 2022, Springer Nature.

Another approach involves incorporating HMPs into a continuous matrix rather than utilizing them alone. HMP-incorporated scaffolds are typically produced through a multistep process using a conventional “top-down” approach, wherein the HMPs are mixed with the bulk scaffold matrix to form the final construct. This method aims to achieve the controlled delivery of bioactive factors, carry cells with high viability, and provide porosity to facilitate oxygen and nutrient exchange [185]. As a proof of concept, Kamperman et al. demonstrated the utilization of single-cell-encapsulated HMPs as building blocks for modular tissue engineering and mixed them with various hydrogel precursor solutions, including agarose, PEGDA, dextran–tyramine, collagen, alginate/GelMA, and alginate [186] (Figure 5A). The resulting HMP-based bioinks were processed using diverse biofabrication techniques, such as extrusion bioprinting, wet spinning, injection molding, emulsification, and photolithography, wherein the cells exhibited over 70% viability.

For bone regeneration, microspheres have been reported to be delivered within a carrier gel to facilitate their transportation to the defect site. For example, Annamalai et al. incorporated MSCs into a chitosan–collagen matrix to create modular tissues, and particles were embedded into fibrin gels to fill critical-size defects in mice [187]. The microtissues within the carrier gel exhibited complete healing, particularly when the MSCs were utilized in a pre-differentiated form (Figure 5B). In a similar investigation, the GelMA hydrogel precursor served as the carrier for electrosprayed MSC-laden GelMA microgels designed for bone regeneration [188]. This innovative approach was subsequently applied to the in situ cranial repair of rats using bioprinting techniques. The assessment through Micro-computed Tomography (Micro-CT) revealed a pattern of new bone formation extending from the periphery toward the center of various defect morphologies, showing the feasibility of the HMP-based 3D bioprinting approach (Figure 5C). In a different study, the sequential release of BMP-2 and BMP-7 growth factors, loaded into poly-electrolyte complexes consisting of poly(4-vinyl pyridine) (P4VP) and alginic acid microspheres, was successfully accomplished within PLGA scaffolds [189]. This approach exhibited the improved osteogenic differentiation of MSCs when both factors were co-administered.

Injectable scaffolds incorporating HMPs are gaining attention in bone regeneration due to the minimally invasive nature of this technique. For example, Yan et al. engineered gelatin HMPs loaded with tetracycline hydrochloride (TH) and incorporated them, along with hydroxyapatite, into alginate-based injectable gel scaffolds [190]. The resulting HMP-reinforced scaffolds exhibited improved mechanical strength, the controlled release of bioactive factors, and significantly higher viability of osteoblasts seeded in the composite gel compared to scaffolds without HMPs.

HMPs can be seamlessly integrated into solid structures, including those crafted through advanced 3D-printing techniques. In a recent study, Zhuang et al. showcased a sustained BMP-2 release from core–shell HMPs with heparin-coated polylactic acid (PLA) cores fabricated through emulsion and alginate shells via electrospraying [191]. These HMPs were integrated into 3D-printed polycaprolactone (PCL) scaffolds, demonstrating their ability to promote osteogenesis both in vitro and in vivo. This highlights the effectiveness of hybrid systems employing both scaffolds and HMPs.

## 5. Conclusions

Hydrogel microparticles (HMPs), fabricated through diverse techniques, emerge as versatile entities, contributing to drug and cell delivery, scaffold production, and bioink development for 3D printing. Their injectability for minimally invasive delivery, coupled with modular properties when composed of different populations, marks them as key players in advancing bone tissue engineering. This injectability characteristic introduces the potential for delivering microspheres multiple times, thereby enhancing current clinical approaches. The adaptability of HMPs for modifications adds to their attractiveness. For instance, beyond their capacity to incorporate antibiotics, HMPs can be designed to be entirely degradable, featuring a cellular “safety switch” that induces cellular apoptosis in carrier cells before hydrogel degradation. The biomaterial can then undergo selective degradation through the inclusion of specific protease sites, allowing tunable degradation after the injury has healed [192].

In the broader context, microspheres exhibit the potential not only to create three-dimensional scaffolds but also to seamlessly integrate into scaffold matrices for bone regeneration. Despite extensive research in this domain, the development of a flawless system capable of fully replacing bone tissue remains an ongoing endeavor. The intricate complexity of bone tissue emerges from its interactions with the surrounding tissues. By leveraging various HMPs to create multiple microenvironments, it becomes possible to generate spatiotemporal microgradients, fostering the formation of functional tissues. Additionally, single-cell encapsulation enables high-throughput screening at the individual-cell level, providing insights into cellular responses to these diverse environments. Scaffolds utilizing microspheres, particularly functional ones serving as molecular carriers, present promising avenues for advancements in bone tissue engineering. The multifaceted attributes of HMPs, ranging from their injectability and degradability to their suitability for the delivery of cell and biological factors, position them as valuable tools in the evolving landscape of bone regeneration research and clinical applications.

## Data Availability

Not applicable.
